# Factors associated with retention intentions among Isibindi child and youth care workers in South Africa: results from a national survey

**DOI:** 10.1186/s12960-018-0307-7

**Published:** 2018-08-29

**Authors:** Tonya R. Thurman, Tory M. Taylor, Johanna Nice, Brian Luckett, Myra Taylor, J. D. Kvalsvig

**Affiliations:** 1 Highly Vulnerable Children Research Center (HVC-RC), Tannery Park, 23 Belmont Road, Rondebosch, Cape Town, South Africa; 2 Tulane International, LLC, Tannery Park, 23 Belmont Road, Rondebosch, Cape Town, South Africa; 3 Tulane University School of Social Work, 127 Elk Place, New Orleans, LA 70112 USA; 4 Tulane University School of Public Health and Tropical Medicine, 1440 Canal St, New Orleans, LA 70112 USA; 5 Discipline of Public Health Medicine, University of KwaZulu-Natal, 236 George Campbell Building, Howard College Campus, King George V Avenue, Durban, 4041 South Africa

**Keywords:** South Africa, HIV and AIDS, Child and youth care worker, Community health worker, Self-efficacy, Retention

## Abstract

**Background:**

Child and youth care workers (CYCWs) are a crucial and growing component of South Africa’s national response to HIV and AIDS and other issues affecting children and families. CYCWs use the community-centred Isibindi model of care to reach the most vulnerable with key services including psychosocial, health, economic and education support. Like others in similar professions, they may be at risk for occupational challenges affecting retention.

**Methods:**

This study uses data from the first nationally representative survey of CYCWs in South Africa to identify factors associated with workers’ retention intentions. Data were collected in 2015 as part of a formative evaluation conducted around the mid-point of a nationwide Isibindi programme expansion. A total of 1158 CYCWs from 78 sites participated. The response rate for the sample was 87%. Questions addressed demographics, work history, retention intentions, training, mentorship and supervision experiences, workload and remuneration. Mixed effects regression models with random intercepts for project site and mentor were used to estimate factors associated with retention intentions.

**Results:**

High-quality mentorship and frequent supervision support retention intentions among CYCWs. Respondents who indicated that wanting to help children or the community was their primary motivator for seeking work as a CYCW were also more likely to report intending to continue working as a CYCW. High perceived workloads and feeling threatened or unsafe on the job were negatively associated with retention intentions. As CYCWs gained experience, they were also less likely to intend to stay.

**Conclusions:**

Understanding the factors affecting retention in the CYCW workforce is vital to helping vulnerable children and families across South Africa access key social and health services. Findings highlight the importance of mentoring and supervision as part of the Isibindi model and the value of support for manageable workloads, workplace safety, and career advancement opportunities for promoting worker retention.

## Background

Community workers form an essential component of social and health service human resources in South Africa. The country’s community-based child and youth care workers (CYCWs) use the Isibindi model of care to serve vulnerable children around the country [1], with a principal focus on providing support to the more than 2 million children orphaned or otherwise affected by HIV and AIDS [2]. Services provided range from HIV prevention and healthcare referrals to psychological support, education assistance, child protection, linkages to social grants, and more [3]. Isibindi was formally developed in 2005 after related activities dating back to 2000, by the country’s National Association of Child Care Workers (NACCW). In September 2012, the programme began a major 5-year expansion with support from the government of South Africa’s Department of Social Development (DSD) and the U.S. Agency for International Development (USAID) through the President’s Emergency Plan for AIDS Relief (PEPFAR).

Working from more than 300 Isibindi sites and counting, CYCWs are recruited from the communities they serve and receive pre-service and ongoing training and support preparing them to work effectively with children and families during home visits and through special programming such as Safe Parks, disability services and early childhood development [1]. Community-based organisations implement and manage project operations at Isibindi sites, while NACCW continues to plan and provide accredited training for CYCWs and to hire and supervise the mentors who work closely with them. CYCWs’ initial training is completed during their first 2–3 years of programme engagement and includes 14 core competency modules and two fundamental competency modules each lasting approximately 6–30 h, with 30% of training classroom-based and 70% taking place on the job. Training covers areas such as human rights, child care, child development, activity programming, observing and reporting, interpersonal relationships and team building. Isibindi CYCW trainees were receiving official monthly stipends of R1,320 and R2,100 in their first and second years respectively at the time of this report (approximately US$94 and US$149 at current exchange rates), with payment minimums increasing annually for inflation. Salary levels following training vary widely between sites [4].

Previous studies have found that Isibindi programming is associated with positive outcomes for children and adolescents including increased access to social grants and basic material resources [3, 5] as well as lower HIV risk behaviour [6]. Evidence from qualitative research, however, suggests that these results may come at a high personal cost to Isibindi CYCWs in South Africa, who report experiencing work-related stress, secondary trauma, difficult working conditions, and poor stakeholder relations [7]. These findings echo those from other studies of community workers in low-income country settings whose job responsibilities may overlap with those of CYCWs. A mixed methods study from South Africa, for example, found that lay HIV counsellors experienced high levels of emotional exhaustion [8]. Ethnographic research conducted among community health workers (CHWs) in Mozambique and Ethiopia also found that psychosocial and interpersonal issues including job-related frustration and stress were prevalent [9]. Burnout, job dissatisfaction, and limited social support are among the strongest predictors of turnover or intention to leave among child welfare and other human service workers in the United States [10], and a review of health interventions in sub-Saharan Africa highlighted low remuneration and workers’ mistreatment as drivers of attrition there [11].

Despite the reliance on CYCWs as frontline service providers for vulnerable children and families in South Africa, little is known about their wellbeing or the factors that may limit the effectiveness and sustainability of Isibindi programming. This study presents results from a nationally representative survey of CYCWs working at 78 community project sites in South Africa, including factors associated with respondents’ intentions to stay employed in their current capacity for at least the next 12 months. Specific attention is given to identifying modifiable aspects of project planning and implementation that may reduce staff turnover and promote continuity in service delivery to children and families.

## Methods

Data were collected through a survey of CYCWs conducted from April through June 2015 as part of a broader formative evaluation of the Isibindi programme, at approximately the halfway point in a major expansion designed to train and deploy 10 000 additional CYCWs at new sites across the country [12]. A cross-sectional design was employed with individuals in the sample selected to represent all 4834 CYCWs employed at 318 Isibindi sites in total. The sample frame was based on a list of project sites with CYCW population size estimates provided by NACCW. Sites were stratified by pre-post expansion establishment status, with 83 sites established before 2012 and 235 sites established in 2012 or later. Within each time-of-establishment substratum, 40 sites were chosen with probability proportionate to size, with the size being the number of CYCWs and distribution across all provinces. All 1342 CYCWs employed at the selected sites were invited to complete an anonymous, self-administered, paper-based questionnaire in English. A total of 1158 CYCWs took part (86% response rate). Two selected project sites failed to submit any questionnaires, yielding 78 sites in the analytic sample.

### Outcome measure

#### Retention

CYCWs were asked, “Within the next 12 months, do you think you will look for a new job with another employer?” Those who answered “no” (as opposed to “yes” or “maybe”) were categorised as intending to stay.

### Predictor measures

#### Emotional exhaustion

Participants were asked to complete a modified version of the Maslach Burnout Inventory Human Services Scale (MBI-HSS) that included measures adapted from the emotional exhaustion subscale, such as “My work tires me emotionally” and “I feel frustrated by my work” [13]. The MBI-HSS has been used in South Africa among registered nurses with good internal and external validity [14]. Response options were coded 1 for “rarely,” 2 for “sometimes,” and 3 for “often”—and summed to create the total score (Cronbach’s alpha = 0.61).

#### Mentorship

CYCWs were asked, “How often does your mentor’s help and advice influence your day to day work?” “How often does your mentor treat you with respect?” and “In general, how comfortable do you feel asking your mentor for help with work-related concerns?” Responses of “always or often” (versus “sometimes” or “rarely or never”) on the first two items and “very comfortable” on the third item (versus “somewhat” or “not at all”) were considered indicative of high-quality mentorship and used to generate a binary variable.

#### Supervision

Supervision was measured using four items addressing the CYCW’s experience during the last 3 months: “How often did you meet with your [mentor]/[team leader] in a group with other CYCWs to discuss your work?” and “How often did you and your [mentor]/[team leader] meet in person—only the two of you?” Response options included “never” (0), “less than once a month” (0.5), “once a month” (1), and “more than once a month” (2). Coded values were summed to estimate the monthly number of supervisory interactions.

#### Motivation

Respondents were asked to select the “most important reason that [they] became a CYCW” from a list of six structured response options (plus other/specify). Those who responded that they became a CYCW to help their community or to work with children and youth, instead of for personal gain, were dichotomously categorised as altruistically motivated.

#### Perceived workload burden

Respondents were asked to indicate whether they felt their workload was “…too high, I have too much work to do a good job with all of it,” “…okay, I am busy but I can meet all my responsibilities,” or “…too small, I can take on more work and still do a good job.” Those who responded that their workload was too high were categorised as having a high burden.

#### Unsafe conditions

Respondents were asked to indicate if they had ever felt threatened or unsafe while doing their job as a CYCW (yes/no).

#### Compensation problems

CYCWs were asked how much they are supposed be paid per month, how much pay they received the previous month, and whether they had received that payment on time. Those who reported receiving no pay, less than their expected pay in the previous month, or having been paid late were categorised as having compensation problems.

#### Training modules completed

Respondents were asked to indicate every core training module they had completed, using a list. This measure reflects the number of core modules completed (out of 14 maximum).

### Statistical analyses

Nationally representative sampling weights were calculated from the sample frame data provided by NACCW and the numbers of completed questionnaires received. Regression models were estimated at the individual CYCW level with clustering at the project/site and mentor levels, with CYCWs nested within project sites and project sites nested within mentors. Records with a single missing response on the emotional exhaustion scale items had its value imputed (*n* = 79), whereas those with more than one missing response were excluded (*n* = 33). Records without information on the CYCWs’ duration of employment were dropped from the model (*n* = 75). Records with missing data for dichotomous variables were coded to reflect the converse of the condition indicated.

Descriptive statistics were estimated using SAS v9.3 (Carey, NC) and adjusted to account for sampling weights. Mixed effects logistic regression using Stata IC 14 (College Station, TX) was utilised to estimate odds ratios associated with factors predictive of intention to remain employed as a CYCW. Respondents’ age, gender, length of time spent working as a CYCW, and high school completion status were included in the model. Random intercepts for project site within mentor were included to correct standard errors for clustering at those levels. Intraclass correlations were estimated at the sampling strata levels of province and established/new project site and found to be negligible after accounting for clustering by project site and, therefore, not accounted for in the multilevel model. Although the intraclass correlation for mentors was below 10% in the model, mentor-level random intercepts were included to enable comparisons of the amount of variance explained at the mentor as well as project site levels.

## Results

Table 1 displays the unweighted characteristics of survey respondents and the nationally representative weighted frequencies and means for all variables included in the model. CYCWs who intended to remain in their current position for at least the next 12 months comprised 46% of the weighted sample. More than 90% of CYCWs reported that their principal motivation to become a CYCW was an altruistic one. Almost 40% felt that their workload was too high and about half (53%) had experienced an unsafe situation at work. Over 60% had experienced a compensation problem in the last month. Two thirds reported receiving high-quality mentorship.

**Table 1:** Unweighted and weighted frequencies and means of CYCW characteristics

	Intention to remain as CYCW			
	Unweighted (*N* = 1 158)		Weighted (*N* = 4 834)	
Variable	*N*	%	*N*	%
Male gender	195	16.9	748	15.5
Did not finish high school	277	23.9	1 213	25.1
Became CYCW to help people	1 051	90.8	4 411	91.3
Feels work load is too high	449	38.8	1 851	38.3
Experienced unsafe situation working as CYCW	608	52.6	2 592	53.6
Not paid, under paid or paid late last month	707	61.1	2 966	61.4
Feels mentor is helpful, open and respectful	747	64.6	3 144	65.0
Intends to remain working as a CYCW next year	556	48.1	2 220	45.9
	Mean	SE	Mean	SE
Age	31.9	0.2	32.0	0.5
Years working as a CYCW	2.9	0.1	3.1	0.2
Emotional exhaustion subscale	13.6	0.1	13.7	0.1
Meetings with mentor and team leader per month	5.0	0.1	5.1	0.1
Number of training modules completed	11.1	0.1	11.5	0.3

Table 2 presents the results of the mixed effects logistic regression modelling intention to remain a CYCW. Each additional year of age increased the odds that a CYCW would intend to stay by 6%, but each additional year of employment as a CYCW decreased these odds by 13%. High workload burden reduced the odds of intending to stay by 34%, while experiencing an unsafe situation at work reduced the odds by 36%. Each additional monthly interaction with mentors and team leaders (e.g. supervision) increased CYCWs’ odds of intending to stay by 14%. Quality mentorship increased the odds of intending to stay by 77% and becoming a CYCW for principally altruistic reasons nearly tripled the odds of intending to stay. Emotional exhaustion was not associated with retention intentions nor were compensation problems or training. Nearly 30% of the variance in retention intentions was explained at the project site level, while less than 6% was explained at the mentor level.

**Table 2:** Mixed effects logistic regression predicting intention to remain a CYCW for the next 12 months (*N* = 1 050)

Variable		95% CI		Log odds
	Odds ratio	Lower	Upper	*p* value
Age	1.06	1.03	1.08	0.001
Male gender	0.93	0.62	1.41	0.737
Did not finish high school	1.08	0.72	1.62	0.698
Years working as a CYCW	0.87	0.79	0.95	0.003
Became CYCW to help people	2.80	1.56	5.00	0.001
Emotional exhaustion	1.00	0.93	1.07	0.961
Feels work load is too high	0.66	0.47	0.93	0.017
Not paid, under paid or paid late last month	1.09	0.71	1.67	0.704
Experienced unsafe situation working as CYCW	0.64	0.46	0.88	0.006
Meetings with mentor/team leader per month	1.14	1.03	1.25	0.009
Feels mentor is helpful, open and respectful	1.77	1.26	2.49	0.001
Number of training modules completed	0.95	0.90	1.01	0.135

## Discussion

To our knowledge this is the first study to provide a generalizable profile of Isibindi CYCWs in South Africa, including estimates of workers’ retention intentions. About half of the CYCWs in this study indicated that they intended to look for a new job with another employer in the next 12 months. Quality mentorship was positively associated with retention intentions; however, increasing years of experience as a CYCW had the opposite effect. Care workers who reported receiving frequent supervision had greater odds of intending to stay—as did those who reported becoming a CYCW primarily for altruistic reasons. Those who perceived their workloads as too high or who indicated ever feeling threatened or unsafe while performing their job also had lower odds of intending to stay. Use of these findings to guide CYCW recruitment and support strategies has the potential to maximise the quality and sustainability of the programme.

The Isibindi expansion has been characterised by efforts to retain at scale the intensive CYCW training and mentorship model originated and led by NACCW. Notably, results from the present analysis confirm the importance of mentorship for workers at new and established sites countrywide. CYCWs who rated their mentors as influential, approachable and respectful had greater odds of intending to remain in their jobs. This aligns with other evidence suggesting that the quality of supervision, even more than its frequency, is pivotal for retention among CHWs [15]. In the present study, CYCW’s intentions to stay were further bolstered by increasing numbers of monthly supervision meetings with their mentor or team leader. The salience of supervision frequency in this context might be explained by CYCW’s high levels of satisfaction with these interactions.

While the influence of training on retention intentions was less clear, prior research among CHWs suggests that training is linked to retention in similar contexts. For example, a study of volunteer CHWs from Uganda noted that the programme’s “interactive learning pedagogy and emphasis on community development and facilitation skills combined with regular contact and training by supervisors likely served as retention promoting, non-financial motivators,” and workers in the study ranked education/training second-highest among seven employment motivating factors ([16], p394). A case study of a targeted training program for community doctors practicing in rural areas of Mali likewise found that the training contributed to retention [17]. Training was reflected in the current analysis uni-dimensionally (as the number of core training modules completed), and other aspects of training may hold greater significance for retention among CYCWs. Future research examining training outcomes for this population should encompass a range of factors such as the type and duration of training as well as participants’ reflections on its value, quality, and relevance to their work.

A majority of CYCWs in this study were motivated to become care workers for altruistic reasons, and they were significantly more likely to intend to stay in their positions than those who cited practical or personal advancement purposes for joining. Evidence from qualitative research conducted with community health workers in Ethiopia and Mozambique provides additional support for community- and family-based socio-moral values as primary drivers of workers’ retention intentions [9]. In a study from Bangladesh, CHWs likewise cited feeling needed by the community as underlying their intentions to stay—and the desires for self-development and improving community health as motivating them to become CHWs [18]. The high prevalence of altruism as a motivation among CYCWs suggests the programme is already effectively recruiting those with humanitarian objectives. Accentuated efforts to raise trainees’ awareness of the social value of their work, developing mechanisms to sustain this awareness throughout employment, and/or designing promotional campaigns that stress the programme’s humanitarian achievements could serve as additional motivation for sustained employment.

Unsurprisingly, CYCWs who perceived their workloads as unacceptably high or who had felt threatened or unsafe while doing their jobs exhibited lower intentions to stay. However, compensation problems were not associated with retention intentions. These findings contrast with those from a 2016 review of health intervention sustainability in sub-Saharan Africa, which concluded that low remuneration contributed to workers’ attrition [11]. In qualitative interviews conducted with CYCWs as part of the larger formative evaluation encompassing this national survey, participants also indicated that payment irregularities are common and provoke widespread discontent [12]. Limited alternative work opportunities, a sense that payment schedules would improve, and/or the hope that they would be promoted to higher paying positions as trainers or mentors might contribute to the lack of association of compensation problems with retention intentions. Compensation problems examined in the survey were also largely limited to past-month issues, which may not reflect longer-term or intermittent problems. Finally, the population of active CYCWs may disproportionately include those with the means or will to remain employed despite compensation problems.

The relative insignificance of demographics (including gender and educational attainment) echoes research from other countries pointing to organisational and administrative factors as more influential than personal ones [10, 19] and suggests that Isibindi is readily replicable without narrow targeting of CYCW candidates. At the same time, to qualify as trainees CYCWs must have completed tenth grade and demonstrate basic written and spoken English, prerequisites which could hinder scalability in contexts where these attributes are uncommon among candidates. Findings do however denote that recruitment need not be limited to females for CYCW positions, although additional studies would be useful to identify factors that may promote retention and other desired outcomes in male versus female workers. The fact that intentions to stay were higher among older CYCWs echoes results from a study of CHWs from rural Ghana [20]. This may reflect older CYCWs’ greater commitment to the programme, more limited career mobility aspirations, perceptions that alternative positions for older workers are in short supply, or other factors. Nevertheless, Isibindi sites may wish to make special efforts to recruit older candidates as a means of minimising workforce turnover.

Age notwithstanding, as CYCWs acquire more experience in their positions, they appear more likely to consider alternative employment. Workers’ intentions to stay or leave might reflect several motivating factors unrelated to dissatisfaction—such as personal or family considerations, upward mobility and career advancement. Future studies examining retention among CYCWs may benefit from asking those who intend to leave what their reasons are and if they plan to remain in the child and youth care work sector. The programme should seek to incentivise retention through continued provision of consistent support to workers, but also offer career paths within the organisation that capitalise on human resource investments and help to retain qualified, experienced CYCWs.

This study has several important limitations including the inability to infer causal relationships due to the cross-sectional design. Many of the measures used were ad hoc and have not been formally tested. Further, retention intention was used as proxy for actual retention. Other researchers have noted the need for rigorous studies of lay health workers’ retention, as well as the challenges associated with generating such measurements [21]. In the present study, the integrity of the sampling frame and accuracy of weighted frequencies used may also have been influenced by unknown fluctuations in CYCW staffing. Self-administered questionnaires might also be subject to variability in respondents’ comprehension of the questions and format, contributing to missing data and potential response bias. Predictive models of missing data in the emotional exhaustion subscale demonstrate that older CYCWs were more likely to omit responses. Although the model was adjusted for age, this non-randomness in item nonresponse may affect the model estimate. Additionally, even the limited imputations applied here may bias the model estimate towards the mean or null.

## Conclusions

This study offers the first-ever generalizable description of a critical and growing workforce, as well as new evidence about the factors associated with CYCWs’ intentions to continue serving in their positions. Continuity in the delivery of key services to the children and families under CYCWs’ care in turn is expected to promote programme effectiveness. As Isibindi concludes a major national scale-up effort involving hiring, training, deploying and providing long-term professional support to approximately 10 000 CYCWs at recently established sites nationwide, understanding the factors affecting retention in this cadre of workers is vital. Findings strongly support the importance of mentorship and supervision for the sustainability of this decentralised programme model.

To optimise retention among CYCWs, greater efforts are needed to ensure manageable workloads, perceived workplace safety, and meaningful opportunities for professional advancement. Findings suggest that initiatives aimed at developing a stable workforce should target both male and female CYCW candidates and those with primarily humanitarian motives, and/or build workers’ awareness of Isibindi’s many positive effects on children and communities. Finally, a large proportion of the variance across outcomes was explained by site, denoting the potential importance of site-level approaches to project planning, problem identification and response. Future research designed to enable site-level or other disaggregated characterisations and comparisons would be useful to guide critical aspects of projects’ implementation, including CYCW recruitment, hiring and support provision. We hope that these recommendations can be applied by programme implementers to improve service delivery for vulnerable children and their families in South Africa and beyond, and that future research will continue to examine ways to increase and sustain the effectiveness of this and similar community care models.

## Abbreviations

AIDS: Acquired immune deficiency syndrome
CHW: Community health worker
CYCW: Child and youth care worker
DSD: Department of Social Development
HIV: Human immunodeficiency virus
NACCW: National Association of Child Care Workers
PEPFAR: President’s Emergency Plan for AIDS Relief
USAID: United States Agency for International Development
